# Administration-order effects in head-to-head comparisons of the EQ-5D-3L and EQ-5D-5L descriptive systems: evidence from a randomised general-population panel in Great Britain

**DOI:** 10.1007/s11136-026-04419-1

**Published:** 2026-10-22

**Authors:** Ling Jie Cheng, Xun Li

**Affiliations:** 1 National Perinatal Epidemiology Unit, Nuffield Department of Women’s & Reproductive Health, University of Oxford, New Radcliffe House, Radcliffe Observatory Quarter, Woodstock Road, Oxford, OX2 6GG UK; 2 Alice Lee Centre for Nursing Studies, Yong Loo Lin School of Medicine, Centre for Translational Medicine, National University of Singapore, Block MD6, Level 5, 14 Medical Drive, Singapore, 117599 Singapore; 3 School of Engineering, Computing, and Mathematics, Oxford Brookes University, Oxford, UK

**Keywords:** EQ-5D-3L, EQ-5D-5L, Ceiling effect, Administration order, Response contamination, Survey design

## Abstract

**Purpose:**

The EQ-5D-3L and EQ-5D-5L are commonly compared within the same questionnaire, but answering one may influence responses to the other. We randomised administration order to estimate descriptive properties before exposure to the alternative version and quantify order effects.

**Methods:**

In Wave 11 of the Understanding Society Innovation Panel, respondents were randomised to the EQ-5D-5L-first condition (*n* = 952), the EQ-5D-3L-first condition (*n* = 961), or the EQ-5D-5L-only condition in the late questionnaire position (*n* = 983). Outcomes included the full-health ceiling, response distributions, and Shannon informativity.

**Results:**

When each version was answered in the early questionnaire position, the full-health ceiling was 52.1% for the EQ-5D-3L and 35.4% for the EQ-5D-5L (difference 16.7% points, 95% CI 12.2 to 21.3), and the EQ-5D-5L was more informative on every dimension. Moving the EQ-5D-5L to the late position without prior EQ-5D-3L exposure changed its ceiling by 2.5 points (95% CI -2.0 to 7.0), whereas prior EQ-5D-3L exposure at the same position raised it by 12.8 points (8.2 to 17.4). Among interviewer-present interviews, the EQ-5D-5L ceiling was 34.5% without prior EQ-5D-3L, 43.0% after the EQ-5D-3L without noticing repetition, and 53.2% among those who noticed.

**Conclusion:**

The EQ-5D-5L order effect arose predominantly from prior EQ-5D-3L exposure rather than questionnaire position and was stronger among respondents who noticed the repetition, consistent with response contamination. Studies comparing instruments within one questionnaire should randomise and report administration order.

**Supplementary Information:**

The online version contains supplementary material available at https://doi.org/10.1007/s11136-026-04419-1.

## Abstract (plain language summary)

The EQ-5D is a short questionnaire widely used to measure people’s health. Two versions exist: an older one with three answer options per question and a newer one with five. Researchers usually compare them by asking the same people to complete both in one survey, but answering one version may change how people answer the other. This matters because such comparisons guide decisions about which version to use. Using a British survey that randomly varied which version people answered first, we measured how each version performs free of this influence. The newer version described health in more detail. However, when people answered the newer version after the older version, many more reported full health, reducing the apparent difference between the two versions. This pattern was strongest among people whom the interviewer noticed spotting that the same questions were being asked twice, which is consistent with the repetition influencing subsequent responses. Studies comparing questionnaires within one survey should vary the order at random, or at least report it.

**Supplementary Information:**

The online version contains supplementary material available at https://doi.org/10.1007/s11136-026-04419-1.

## Introduction

The EQ-5D is the most widely used generic preference-based measure of health-related quality of life (HRQoL) [1–3]. The adult EQ-5D questionnaires comprise a descriptive system covering five dimensions (Mobility, Self-care, Usual activities, Pain & discomfort, and Anxiety & depression) and the EQ VAS. When a country-specific value set is applied, each EQ-5D profile described by the descriptive system is assigned an EQ-5D value for use in economic evaluation and other health outcomes research [4, 5]. The original three-level version, the EQ-5D-3L, classifies each dimension as “no problems”, “some problems” or “extreme problems”. However, the EQ-5D-3L has a pronounced ceiling effect: a large proportion of general-population respondents report full health, limiting sensitivity to mild and moderate health problems. The EQ-5D-5L was developed to address this limitation by adding two intermediate levels to each dimension, with the aim of improving descriptive richness and discriminatory power [6].

A substantial literature compares the descriptive-system performance of the EQ-5D-3L and EQ-5D-5L. Common criteria include feasibility (completeness of response), the full-health ceiling, informativity, meaning how widely responses spread across the available levels, summarised using Shannon’s indices, redistribution of responses from the coarser to the finer scale, and the logical consistency of paired responses [7]. Two systematic reviews and a recent meta-analysis [8–10], alongside primary comparison studies in general and clinical populations across many countries [11–13], have concluded that the EQ-5D-5L reduces the ceiling and improves informativity while retaining feasibility and validity [14]. The EQ-5D-5L’s measurement properties continue to be actively investigated in general populations, including comparisons with other generic instruments such as PROMIS [15], analyses of its composite and component items [16], and comparisons among EQ-5D versions [17]. Administration sequence has varied across head-to-head studies and is not always reported [8, 9].

Less attention has been paid to whether the data structures used in these comparisons provide unbiased estimates of the difference between instruments. Within-person designs are attractive because they hold respondent characteristics constant, but they also rely on an important assumption: that completing one EQ-5D version does not influence responses to the other. This assumption is unlikely to be innocuous. Using a randomised experiment embedded in the Understanding Society Innovation Panel, Hernández-Alava and Pudney [18] demonstrated that the sequencing and timing of EQ-5D-3L and EQ-5D-5L completion significantly alter responses, most clearly for the Pain & discomfort dimension, and change the statistical models used to map between the two versions [19, 20].

The implications for descriptive-system comparisons of the EQ-5D-3L and EQ-5D-5L remain unclear. If prior exposure to one version changes responses to the other, comparisons that pool responses without accounting for administration order may misestimate the ceiling, informativity and other descriptive-system properties they aim to quantify. This matters because the difference in the full-health ceiling between the EQ-5D-3L and EQ-5D-5L is cited both as justification for adopting the EQ-5D-5L and to anticipate how utilities change when instruments are switched [21, 22].

This question requires an explicit measurement model. We assume that each respondent has an underlying health state on each dimension and that a response maps that state onto the available response options [23, 24]. Two mechanisms can then cause EQ-5D-3L and EQ-5D-5L responses to differ. The first is a stable between-version measurement difference: at the same underlying level of health, the two descriptive systems may map respondents to their response categories differently. Differential item functioning provides a related framework for conditional differences in response behaviour [25]. The second is response contamination, whereby answering one version changes the response process applied to the other [18]. The two mechanisms are separable by design rather than by inspection: stable between-version differences persist when each version is answered without prior exposure to the other, whereas contamination appears only after such exposure. On this model, a logically inconsistent pairing is an observable consequence of a mismatch between the two mappings, rather than a defect to be counted in its own right.

This study addresses that gap using the randomised-order EQ-5D experiment in the Understanding Society Innovation Panel. Our objectives were to estimate the descriptive-system properties of the EQ-5D-3L and EQ-5D-5L free of contamination, using each instrument as answered in the early questionnaire position; to quantify how far those properties are biased when order is ignored; to separate survey position from prior exposure to the alternative version; and to test whether any contamination scales with respondents’ awareness of having answered the same questions twice, which would help distinguish contamination from a stable between-version measurement difference.

## Methods

### Data source and experimental design

Data were drawn from Wave 11 of the Understanding Society Innovation Panel, part of the UK Household Longitudinal Study. The Innovation Panel is a probability-based household panel, sampled using a two-stage stratified design and reserved for methodological experiments. Fieldwork was conducted from June to September 2018, and anonymised data are distributed by the UK Data Service (study number 6849). The panel interviews adults aged 16 years and older; interviews lasted approximately 20 min and covered a broad range of socioeconomic topics.

The “Variations of the EQ-5D questions” experiment randomised respondents, at household level, to one of three conditions. The questionnaire contained two fixed EQ-5D positions, an early position about halfway through the interview and a late position about three-quarters of the way through, separated by unrelated modules. Throughout, early and late refer to position within the survey, not to a later visit. In the EQ-5D-5L-first condition, respondents answered the EQ-5D-5L early and the EQ-5D-3L late; in the EQ-5D-3L-first condition, this order was reversed; in the EQ-5D-5L-only condition, respondents answered the EQ-5D-5L late and were routed past the early position entirely, receiving no EQ-5D-3L at any point. The EQ-5D-5L items answered late are the same items in the same place for the EQ-5D-3L-first and EQ-5D-5L-only conditions, which is what allows position and prior exposure to be separated.

All analyses concern the five dimension items and the EQ-5D profile they define. The module did not include the EQ VAS and no value set was applied, so no EQ-5D values were computed. We test the order of the two instruments relative to one another, not the order of items within an instrument, which was fixed.

### Analytic samples

Responses coded as missing, refusal, “don’t know” or inapplicable were treated as invalid. Proxy interviews were not randomised to an EQ-5D condition and contributed no EQ-5D data: 94 of the 2,990 Wave 11 records carried the proxy code, all outside the randomised sample, leaving 2,896 randomised respondents. Proxies therefore fall outside the analytic sample by design rather than through an exclusion step. For the comparison of the two versions we used each instrument as answered in the early position, before the other had been seen; we refer to this as the early-position comparison. Because the two sets of responses come from different randomised arms, it is a between-person comparison of independent groups.

Analyses of response combinations and logical consistency require both versions from the same respondent, so these used participants in the EQ-5D-5L-first and EQ-5D-3L-first conditions who validly answered both and were treated as paired data. Between 1,712 and 1,719 respondents contributed; the number varies by dimension because a respondent missing one item still contributes to the others. The ceiling requires a complete five-item profile, whereas dimension-level analyses require only a valid response to that dimension. Order analyses contrast each instrument answered early with the same instrument answered late. These contrasts are between randomised arms and therefore independent, whereas the within-person comparisons are paired.

### Measurement properties

Feasibility was defined as the proportion of valid responses per dimension. The ceiling was defined as the proportion of respondents reporting full health, profile 11111, and was reported overall and by long-standing illness, age group, and sex. Informativity was summarised for each dimension using Shannon’s index, H′ = −Σp_i_ln(p_i_), where p_i_ is the observed proportion at level i, and Shannon’s venness, J′ = H′/ln(L), where L is the number of response levels, either three for the EQ-5D-3L or five for the EQ-5D-5L [7]. We also counted the number of distinct EQ-5D profiles observed for each version [26]. Feasibility and informativity are calculated at dimension level, whereas the ceiling is calculated only among respondents with a complete five-item profile.

Redistribution was examined by cross-tabulating each respondent’s EQ-5D-3L and EQ-5D-5L level within each dimension, showing how responses at each EQ-5D-3L level were distributed across the finer EQ-5D-5L scale.

Logical inconsistency was defined following Janssen et al. [13] as a paired response that could not be reconciled across the two scales: an EQ-5D-3L level 1 response, indicating no problems, paired with an EQ-5D-5L response of moderate problems or worse; an EQ-5D-3L level 3 response paired with an EQ-5D-5L response of moderate problems or better; or an EQ-5D-3L level 2 response paired with either EQ-5D-5L level 1 or EQ-5D-5L level 5. The inconsistency rate was calculated as the proportion of paired responses meeting any of these criteria and was reported by dimension. In terms of the measurement model above, such a pairing indicates that the two mappings are mutually incompatible for that respondent and dimension, so we treat the rate as a descriptive indicator of mismatch rather than of data quality. 

### Administration order, prior exposure and analysis

To quantify bias arising from unaccounted administration order we compared, for each version, the full-health ceiling and mean level sum score when the instrument was answered early with the values when it was answered late. The level sum score is an unweighted total of the five dimension levels, higher values indicating worse health; its range is 5 to 25 for the EQ-5D-5L and 5 to 15 for the EQ-5D-3L, so level sum scores are compared only within an instrument.

For the EQ-5D-5L, the three-arm design distinguished three conditions: the early position, the late position with no prior EQ-5D-3L exposure, and the late position after the EQ-5D-3L. For the EQ-5D-3L, for which no EQ-5D-3L-only arm was available, we compared the early position with the late position after the EQ-5D-5L. The first two EQ-5D-5L conditions differed only in questionnaire position. By contrast, the latter two differed only in whether the EQ-5D-3L had already been answered, while both occupied the same late position. This contrast therefore estimated the effect of prior exposure with questionnaire position held constant. Together, the two contrasts summed exactly to the total order effect.

The Innovation Panel also records an interviewer observation of whether the respondent noticed being asked the same questions twice, put only to respondents receiving both versions and only where an interviewer was present. Contamination should be stronger among respondents who registered the repetition, whereas stable between-version measurement differences should not depend on awareness of the repetition. We therefore compared the EQ-5D-5L ceiling across respondents with no prior EQ-5D-3L, those answering it after the EQ-5D-3L without noticing, and those observed noticing, restricting all three to interviewer-present interviews and adjusting for age, sex and long-standing illness.

Differences in proportions are reported as risk differences in percentage points with Wald 95% confidence intervals alongside chi-squared tests, using continuity-corrected p values for two-by-two comparisons. Balance across arms used analysis of variance for age and chi-squared tests for categorical characteristics, and differences in mean level sum scores used Welch’s t test. Distributions of response combinations and of levels across conditions were tested by Pearson’s chi-squared; because several cells are sparse we also report a p value from 2,000 permutations of the condition label, and where the same test was applied across the five dimensions we report Holm-adjusted p values. Within-person comparisons used McNemar’s test, and intervals for differences in H′ came from 1,000 bootstrap replicates stratified by arm. Analyses used Stata with a fixed seed.

## Results

### Sample characteristics and response completeness

Of 2,990 Wave 11 Innovation Panel records, 952 respondents were randomised to the EQ-5D-5L-first condition, 961 to the EQ-5D-3L-first condition, and 983 to the EQ-5D-5L-only condition. The remaining 94 were proxy interviews that were not randomised to an EQ-5D condition and contained no EQ-5D data. The randomised arms were balanced on age (one-way analysis of variance *p* = 0.48), sex (*p* = 0.79), and long-standing illness (*p* = 0.24) (Table 1). Nominal differences were observed for economic activity, nation, and self-rated health (all *p* < 0.05). Just over half of respondents were female, the mean age was in the early 50s, and approximately one-third reported a long-standing illness.

**Table 1:** Respondent characteristics by randomised arm

Characteristic	Arm 1: EQ-5D-5L → EQ-5D-3L (*n* = 952)	Arm 2: EQ-5D-3L → EQ-5D-5L (*n* = 961)	Arm 3: EQ-5D-5L only (*n* = 983)	All arms (*n* = 2896)	*p* value
Age, mean (SD), years	51.8 (18.4)	51.3 (18.1)	50.7 (18.4)	51.3 (18.3)	0.480
Sex					0.789
Male	432 (45.4)	424 (44.1)	432 (43.9)	1288 (44.5)	
Female	520 (54.6)	537 (55.9)	551 (56.1)	1608 (55.5)	
Age group					0.873
16–34	194 (20.4)	207 (21.6)	219 (22.3)	620 (21.5)	
35–54	304 (32.0)	313 (32.7)	320 (32.6)	937 (32.4)	
55–74	362 (38.1)	342 (35.7)	344 (35.1)	1048 (36.3)	
75+	90 (9.5)	96 (10.0)	98 (10.0)	284 (9.8)	
Marital status					0.708
Married/cohabiting	603 (63.8)	603 (63.0)	601 (61.4)	1807 (62.7)	
Single, never married	192 (20.3)	198 (20.7)	211 (21.6)	601 (20.9)	
Separated/divorced	102 (10.8)	94 (9.8)	101 (10.3)	297 (10.3)	
Widowed	48 (5.1)	62 (6.5)	66 (6.7)	176 (6.1)	
Highest qualification					0.969
Degree or higher	375 (40.2)	377 (40.2)	388 (40.5)	1140 (40.3)	
A-level or equivalent	186 (19.9)	204 (21.7)	207 (21.6)	597 (21.1)	
GCSE or equivalent	222 (23.8)	217 (23.1)	223 (23.3)	662 (23.4)	
Other qualification	57 (6.1)	55 (5.9)	60 (6.3)	172 (6.1)	
No qualification	93 (10.0)	85 (9.1)	81 (8.4)	259 (9.2)	
Economic activity					0.045
Employed/self-employed	479 (50.3)	516 (53.7)	556 (56.6)	1551 (53.6)	
Unemployed	29 (3.0)	31 (3.2)	40 (4.1)	100 (3.5)	
Retired	294 (30.9)	289 (30.1)	276 (28.1)	859 (29.7)	
Student	50 (5.3)	40 (4.2)	41 (4.2)	131 (4.5)	
Long-term sick/disabled	46 (4.8)	40 (4.2)	43 (4.4)	129 (4.5)	
Other	54 (5.7)	45 (4.7)	27 (2.7)	126 (4.4)	
Nation					0.043
England	817 (85.9)	861 (89.7)	855 (87.0)	2533 (87.5)	
Wales	62 (6.5)	34 (3.5)	55 (5.6)	151 (5.2)	
Scotland	72 (7.6)	65 (6.8)	73 (7.4)	210 (7.3)	
Area					0.655
Urban	729 (76.7)	719 (74.9)	748 (76.1)	2196 (75.9)	
Rural	222 (23.3)	241 (25.1)	235 (23.9)	698 (24.1)	
Long-standing illness, yes	350 (36.9)	321 (33.6)	361 (36.8)	1032 (35.8)	0.235
Self-rated health					0.025
Excellent	126 (14.1)	143 (15.8)	115 (12.4)	384 (14.1)	
Very good	320 (35.8)	340 (37.5)	316 (34.2)	976 (35.8)	
Good	244 (27.3)	253 (27.9)	309 (33.4)	806 (29.6)	
Fair	144 (16.1)	111 (12.2)	121 (13.1)	376 (13.8)	
Poor	60 (6.7)	60 (6.6)	64 (6.9)	184 (6.7)	
SF-12 PCS, mean (SD)	49.0 (11.1)	50.1 (10.5)	49.4 (11.1)	49.5 (10.9)	0.090
SF-12 MCS, mean (SD)	49.0 (10.5)	49.0 (10.6)	48.9 (10.7)	49.0 (10.6)	0.967
Interview mode					0.588
CAPI (interviewer)	531 (55.8)	518 (53.9)	547 (55.7)	1596 (55.1)	
CATI (telephone)	0 (0.0)	0 (0.0)	1 (0.1)	1 (0.0)	
CAWI (web)	421 (44.2)	443 (46.1)	435 (44.3)	1299 (44.9)	

Denominators vary with item non-response (marital status, qualification, nation, urban/rural status, and self-rated health at least 2% missing; SF-12 component scores about 13% missing), so percentages are of non-missing responses and counts may not sum to the column n. Sample is Great Britain (no Northern Ireland in the Innovation Panel). No multiplicity adjustment was applied to the balance tests

*SD *standard deviation, *PCS *physical component summary, *MCS* mental component summary, *CAPI* computer-assisted personal interview, *CATI* computer-assisted telephone interview, *CAWI *computer-assisted web interview

Values are n (%) unless otherwise stated. *p* values test balance across the three randomised arms: one-way ANOVA for continuous variables and Pearson chi-squared for categorical variables

Complete five-item profiles in the early position were available for 881 respondents for the EQ-5D-5L and 884 for the EQ-5D-3L. Completion of the first EQ-5D instrument encountered did not differ significantly across arms (92.5%, 92.0%, and 90.4%; *p* = 0.22). However, after adjustment for age, long-standing illness, qualification, and mode, allocation to the EQ-5D-5L-only arm was associated with non-completion (odds ratio 1.49, 95% CI 1.03 to 2.17). Response and reason codes by arm are reported in Online Resource 1 (Tables S1 and S2).

### Early-position comparison of the EQ-5D-3L and EQ-5D-5L

Feasibility was high and similar between the two versions, with approximately 92.5% of respondents providing a valid response to each dimension. The full-health ceiling was 35.4% (312 of 881) for the EQ-5D-5L and 52.1% (461 of 884) for the EQ-5D-3L, a difference of 16.7% points (95% CI 12.2 to 21.3).

**Fig. 1:**
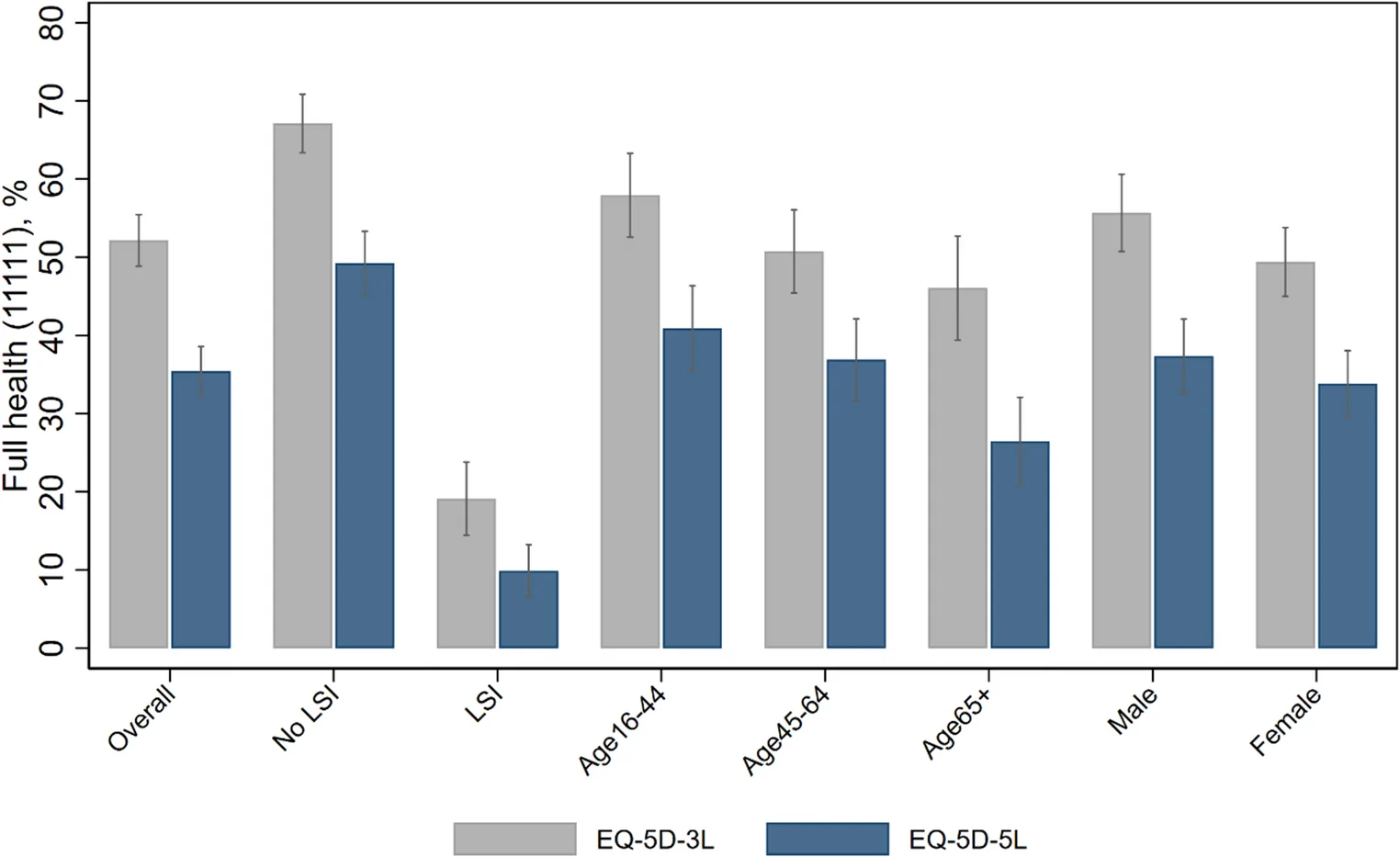
Full-health ceiling by instrument and subgroup, early-position samples. Proportion reporting full health (EQ-5D profile 11111) on EQ-5D-3L and EQ-5D-5L, overall and by subgroup, using each descriptive system as answered in the early questionnaire position before the alternative version was seen. Whiskers are 95% confidence intervals. LSI, long-standing illness

The lower ceiling for the EQ-5D-5L was observed in every subgroup examined (Table 2; Fig. 1), and the EQ-5D-5L recorded more problems on every dimension (all *p* < 0.001; Table S3).

**Table 2:** Full-health ceiling (EQ-5D profile 11111) by subgroup, early-position responses

Subgroup	EQ-5D-3L, *n*/*N* (%)	EQ-5D-5L, *n*/*N* (%)	Difference, percentage points (95% CI)	*p*
Overall	461/884 (52.1)	312/881 (35.4)	16.7 (12.2 to 21.3)	< 0.001
No long-standing illness	408/608 (67.1)	282/573 (49.2)	17.9 (12.3 to 23.4)	< 0.001
Long-standing illness	52/272 (19.1)	30/304 (9.9)	9.2 (3.5 to 15.0)	0.002
Age 16 to 44	190/328 (57.9)	128/313 (40.9)	17.0 (9.4 to 24.7)	< 0.001
Age 45 to 64	172/339 (50.7)	121/328 (36.9)	13.8 (6.4 to 21.3)	< 0.001
Age 65 or older	99/215 (46.0)	63/238 (26.5)	19.6 (10.9 to 28.3)	< 0.001
Male	216/388 (55.7)	149/399 (37.3)	18.3 (11.5 to 25.2)	< 0.001
Female	245/496 (49.4)	163/482 (33.8)	15.6 (9.5 to 21.7)	< 0.001

Cells show the number reporting full health (EQ-5D profile 11111) over the subgroup denominator, with the percentage. Each descriptive system is taken from the randomised arm in which it was answered in the early questionnaire position, before the respondent had seen the alternative version: EQ-5D-5L n=881 and EQ-5D-3L n=884. Confidence intervals for each individual proportion are shown in Fig. 1. The overall relative reduction in the ceiling was 32%

Differences are EQ-5D-3L minus EQ-5D-5L in percentage points with Wald 95% confidence intervals; p values continuity-corrected Pearson chi-squared tests

*CI *confidence interval

### Informativity and observed EQ-5D profiles

**Fig. 2:**
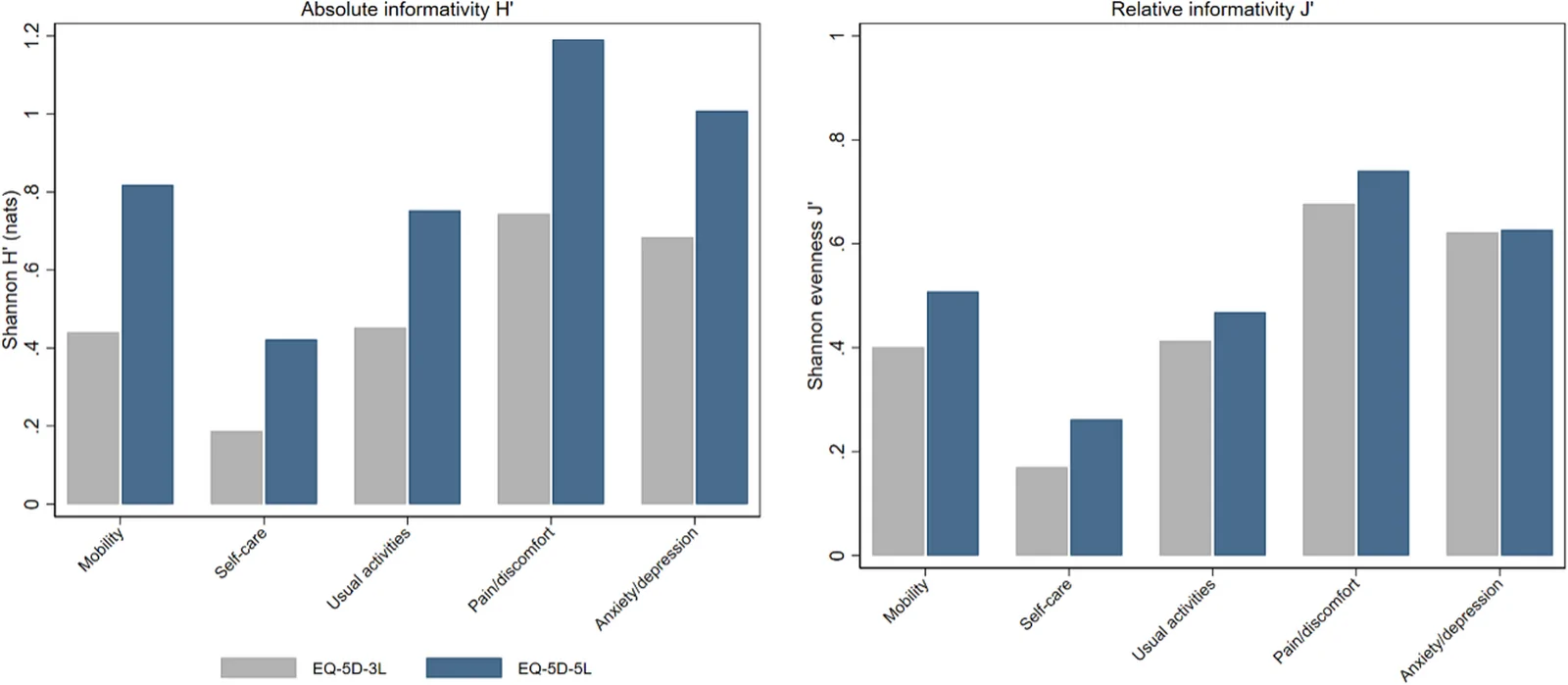
Informativity by dimension and instrument, early-position samples. Shannon’s index H′ and Shannon’s evenness J′ by EQ-5D dimension, using each descriptive system as answered in the early questionnaire position. J′ = H′/ln(L), where L is the number of response levels. Higher values indicate that responses are spread more widely across levels

The EQ-5D-5L had higher Shannon informativity than the EQ-5D-3L on every dimension according to both Shannon’s index H′ and Shannon’s evenness J′ (Table 3; Fig. 2). Mean H′ was 0.84 for the EQ-5D-5L and 0.50 for the EQ-5D-3L, while mean J′ was 0.52 and 0.46, respectively. Respondents used 146 of the 3,125 possible EQ-5D-5L profiles and 52 of the 243 possible EQ-5D-3L profiles.

**Table 3:** Informativity (Shannon’s index H′ and evenness J′) by dimension, early-position responses

Dimension	EQ-5D-3L H′	EQ-5D-3L J′	EQ-5D-3L *n*	EQ-5D-5L H′	EQ-5D-5L J′	EQ-5D-5L *n*
Mobility	0.441	0.401	888	0.818	0.508	882
Self-care	0.187	0.170	890	0.422	0.262	882
Usual activities	0.453	0.413	890	0.753	0.468	882
Pain & discomfort	0.744	0.677	891	1.191	0.740	882
Anxiety & depression	0.684	0.622	888	1.008	0.627	881
Mean	0.502	0.457		0.838	0.521	

H′, Shannon’s index (absolute informativity, nats); J′, Shannon’s evenness (relative informativity, 0 to 1), calculated as H′/ln(L), where L is the number of response levels. Higher values indicate that responses are spread more widely across the available levels. Denominators are dimension-level, that is, respondents giving a valid answer to that dimension, and therefore differ slightly from the complete-profile denominators used for the ceiling in Table 2. Distinct EQ-5D profiles observed: EQ-5D-5L, 146 of 3,125; EQ-5D-3L, 52 of 243. Informativity by administration condition is shown in Fig. 4 and Supplementary Table S8

### Response combinations and logical consistency

Among respondents who answered both versions, most EQ-5D-3L responses indicating no problems corresponded to EQ-5D-5L level 1, whereas responses in the EQ-5D-3L middle category were distributed across the intermediate EQ-5D-5L levels; Pain & discomfort is shown as an exemplar in Table 4. When these cross-tabulations were stratified by administration sequence, the distribution of the 15 possible response combinations differed across all five dimensions and remained different for four after Holm adjustment (Pain & discomfort: chi-squared 91.8, *p* < 0.001). Because several combinations were rare, we repeated each test by permuting the sequence label; permutation p values were similar (Table 4; Table S4). Logical inconsistency was uncommon and did not differ by administration sequence after Holm adjustment (Table S5).

**Table 4:** Redistribution of paired responses from EQ-5D-3L to EQ-5D-5L by administration sequence: Pain & discomfort (exemplar)

Administration sequence	EQ-5D-3L level	EQ-5D-5L = 1	EQ-5D-5L = 2	EQ-5D-5L = 3	EQ-5D-5L = 4	EQ-5D-5L = 5	Total
Both sequences	EQ-5D-3L = 1 (no Pain & discomfort)	947	147	7	1	0	1102
	EQ-5D-3L = 2 (moderate)	29	342	156	31	3	561
	EQ-5D-3L = 3 (extreme)	0	1	3	31	19	54
EQ-5D-3L first	EQ-5D-3L = 1 (no Pain & discomfort)	539	40	1	1	0	581
	EQ-5D-3L = 2 (moderate)	22	174	59	5	0	260
	EQ-5D-3L = 3 (extreme)	0	1	3	16	8	28
EQ-5D-5L first	EQ-5D-3L = 1 (no Pain & discomfort)	408	107	6	0	0	521
	EQ-5D-3L = 2 (moderate)	7	168	97	26	3	301
	EQ-5D-3L = 3 (extreme)	0	0	0	15	11	26

Counts of paired responses among respondents who validly answered both versions (n = 1,717; 869 received EQ-5D-3L first and 848 received EQ-5D-5L first). Pain & discomfort is shown as an exemplar; the other dimensions follow the same general pattern, with most EQ-5D-3L responses of no problems corresponding to EQ-5D-5L level 1 and the broad EQ-5D-3L middle category distributed across the intermediate EQ-5D-5L levels. The distribution of the 15 possible combinations differed by administration sequence for this dimension (chi-squared 91.8, Holm-adjusted p < 0.001) and for three of the other four dimensions; tests for all five dimensions, including permutation p values, are in Supplementary Table S4

### Effects of questionnaire position and prior exposure

**Fig. 3:**
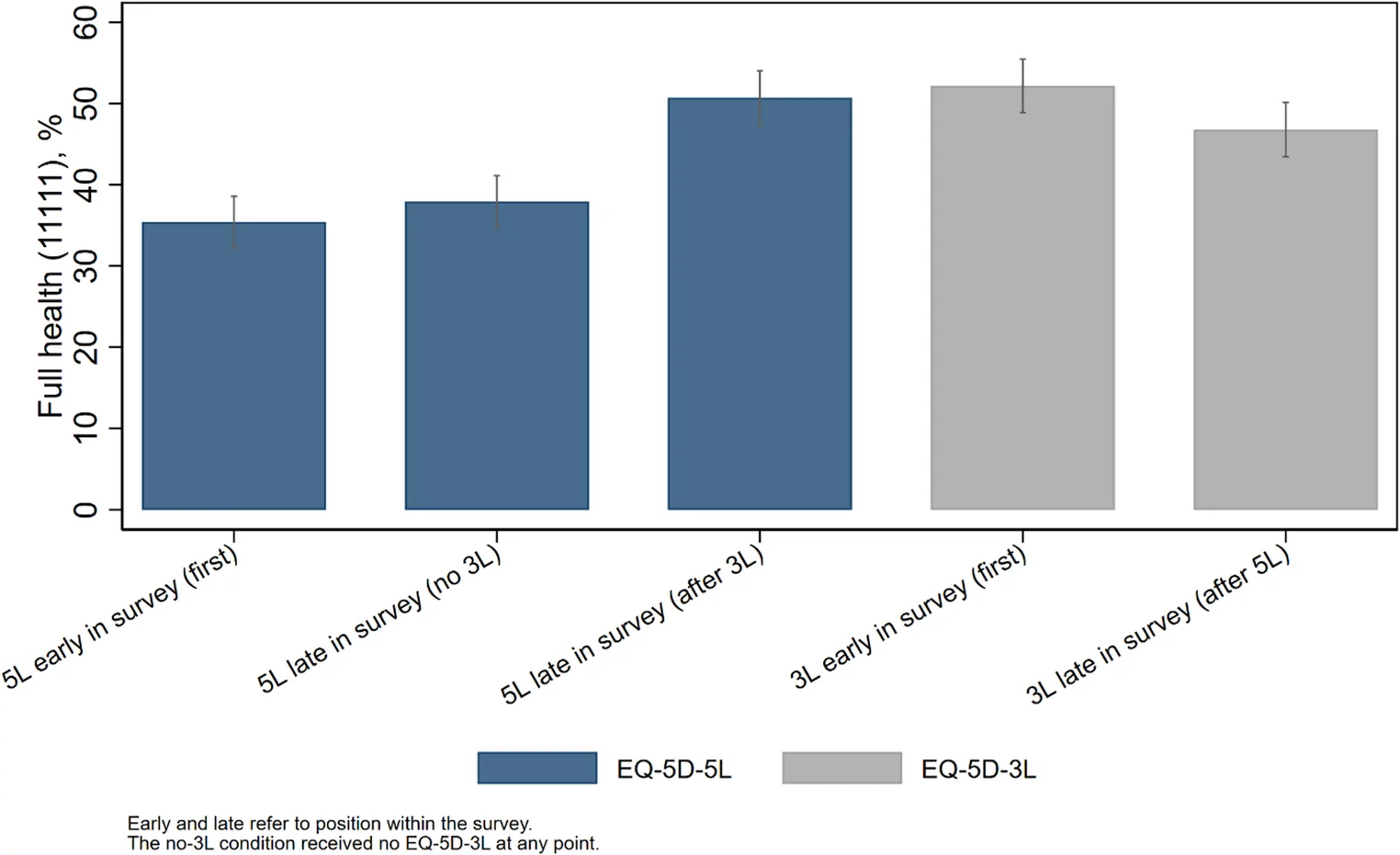
Full-health ceiling by administration position and prior-instrument exposure. Proportion reporting full health (EQ-5D profile 11111) by EQ-5D version and administration condition, with 95% confidence intervals. Early and late refer to position within the survey, not to a later visit. The EQ-5D-5L-only condition received no EQ-5D-3L at any point

The EQ-5D-5L full-health ceiling was 35.4% (95% CI 32.3 to 38.6) in the early position, 37.9% (34.7 to 41.1) in the late position with no prior EQ-5D-3L exposure, and 50.7% (47.4 to 54.0) in the late position after the EQ-5D-3L (Table 5; Fig. 3). Moving the EQ-5D-5L from the early to the late position without prior EQ-5D-3L exposure changed the ceiling by 2.5% points (95% CI − 2.0 to 7.0; *p* = 0.30). At the same late position, prior EQ-5D-3L exposure increased the ceiling by 12.8% points (8.2 to 17.4; *p* < 0.001). The two contrasts summed to a total order effect of 15.3% points (10.7 to 19.9).

**Table 5:** Administration position and prior exposure to the alternative EQ-5D descriptive system

Instrument and condition	Ceiling, *n*/*N* (%)	Difference vs. early position, percentage points (95% CI)	*p*	Mean level sum score (95% CI)
EQ-5D-5L				
Early position	312/881 (35.4)	Reference		7.28 (7.07 to 7.49)
Late position, no prior EQ-5D-3L	337/889 (37.9)	2.5 (− 2.0 to 7.0)	0.299	7.09 (6.90 to 7.29)
Late position, after EQ-5D-3L	442/872 (50.7)	15.3 (10.7 to 19.9)	< 0.001	6.64 (6.45 to 6.82)
EQ-5D-3L				
Early position	461/884 (52.1)	Reference		6.02 (5.93 to 6.12)
Late position, after EQ-5D-5L	399/853 (46.8)	−5.4 (− 10.1 to − 0.7)	0.028	6.26 (6.15 to 6.37)

Early and late refer to position within the survey, not to a later visit. Conditions are defined by the randomised arms: EQ-5D-5L early position = Arm 1; EQ-5D-5L late position with no prior EQ-5D-3L = Arm 3, whose respondents received no EQ-5D-3L at any point; EQ-5D-5L late position after EQ-5D-3L = Arm 2; EQ-5D-3L early position = Arm 2; EQ-5D-3L late position after EQ-5D-5L = Arm 1. The design has no EQ-5D-3L-only arm, so EQ-5D-3L cannot be observed in the late position without prior EQ-5D-5L exposure. Ceiling is the proportion reporting full health (EQ-5D profile 11111). The level sum score is the total of the five dimension levels, with higher values indicating worse reported health; its range is 5 to 25 for EQ-5D-5L and 5 to 15 for EQ-5D-3L, so level sum scores are comparable only within the same version. Differences are in percentage points with Wald 95% confidence intervals and continuity-corrected Pearson chi-squared p values. Moving EQ-5D-5L from the early to the late position without prior EQ-5D-3L exposure changed the ceiling by 2.5 points (95% CI − 2.0 to 7.0); prior EQ-5D-3L exposure at the same late position changed it by 12.8 points (8.2 to 17.4; p < 0.001)

*CI *confidence interval

For the EQ-5D-3L, the ceiling was 52.1% in the early position and 46.8% in the late position after the EQ-5D-5L, a difference of − 5.4% points (95% CI − 10.1 to − 0.7; p = 0.028).

Mean level sum scores showed corresponding changes within each instrument. Moving the EQ-5D-5L to the late position without prior EQ-5D-3L exposure changed the level sum score by − 0.19 (95% CI − 0.47 to 0.10), whereas prior EQ-5D-3L exposure at the late position changed it by − 0.45 (− 0.72 to − 0.19; *p* < 0.001), with lower scores indicating better reported health. For the EQ-5D-3L, the level sum score increased by 0.24 after prior EQ-5D-5L exposure (0.09 to 0.38; *p* = 0.002).

### Order effects on response distributions and informativity

Response-level distributions differed by administration condition on three of the five EQ-5D-3L dimensions after Holm adjustment (Self-care, Usual activities, and Mobility; *p* = 0.001, 0.010, and 0.011, respectively) and on EQ-5D-5L Pain & discomfort (*p* < 0.001), with similar permutation p values throughout (Tables S6 and S7).

**Fig. 4:**
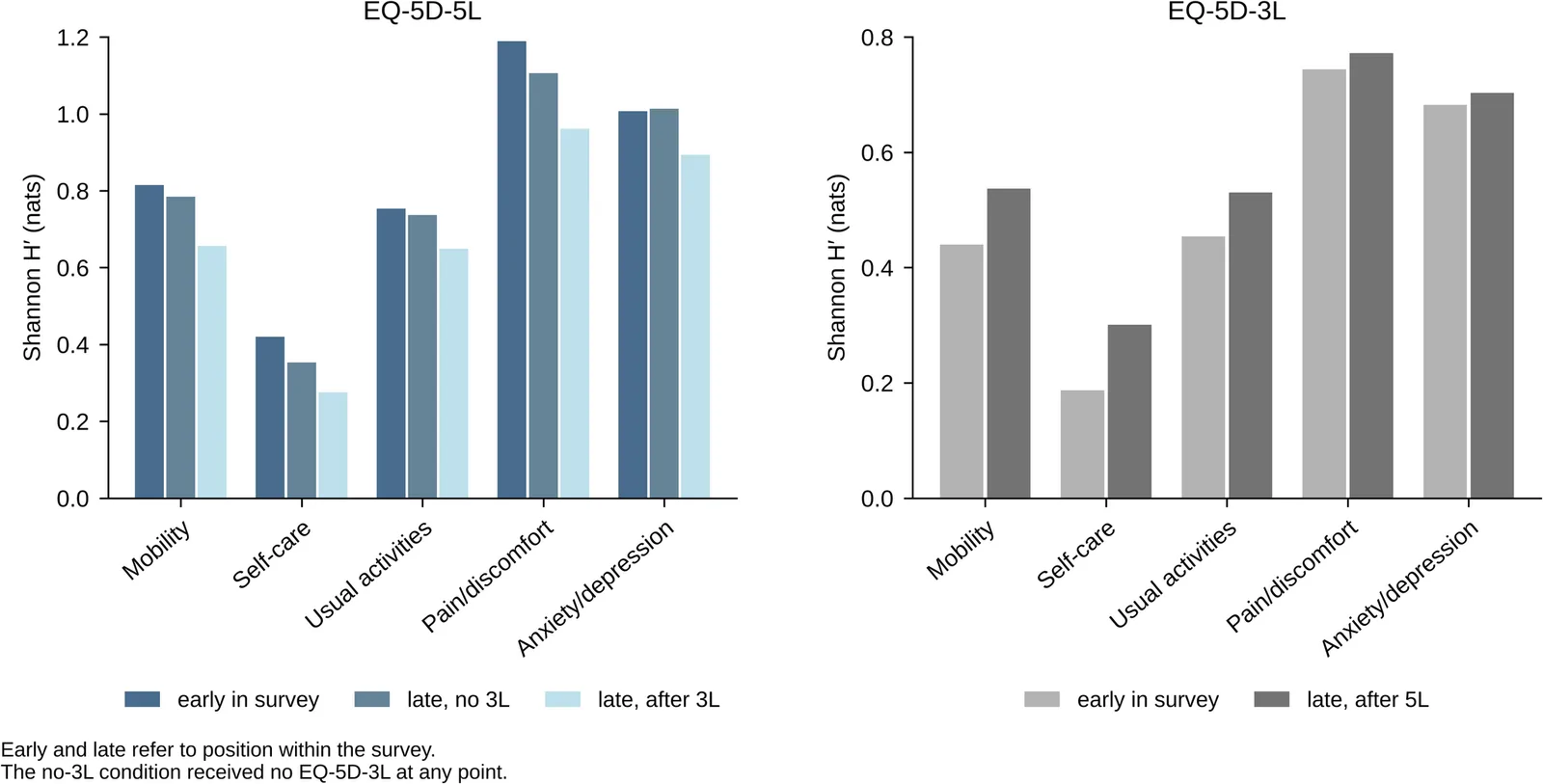
Shannon’s index H′ by dimension and administration condition.Absolute informativity of each dimension for EQ-5D-5L (left) and EQ-5D-3L (right) according to questionnaire position and prior exposure to the alternative version. Early and late refer to position within the survey. The EQ-5D-5L-only condition received no EQ-5D-3L at any point. Higher values indicate that responses are spread more widely across levels

Shannon informativity also varied by administration condition (Fig. 4; Table S8). H′ for the EQ-5D-5L answered after the EQ-5D-3L was lower on all five dimensions, with bootstrap confidence intervals excluding zero. For the EQ-5D-3L answered after the EQ-5D-5L, H′ was higher on three dimensions. The number of distinct EQ-5D-5L profiles was 146 in the early position and 144 in the late position without prior EQ-5D-3L exposure, compared with 113 in the late position after the EQ-5D-3L.

### Fixed-order within-person comparisons

Among the 863 respondents who answered the EQ-5D-3L first, the paired full-health ceiling difference between the EQ-5D-3L and subsequent EQ-5D-5L was 1.3% points (95% CI − 0.4 to 2.9; McNemar *p* = 0.17) (Table S9). Among the 846 respondents who answered the EQ-5D-5L first, the corresponding difference was 11.6% points (9.2 to 13.9). Both estimates were smaller than the early-position between-group difference of 16.7% points.

### Effect of pooling across administration conditions

Using all available data across administration conditions, the estimated difference in the full-health ceiling was 8.2% points (95% CI 5.2 to 11.2), equivalent to 49% of the early-position difference (Table S10). Pooling only the two arms in which respondents received both versions gave a difference of 6.5% points, equivalent to 39% of the early-position difference. The two late-position EQ-5D-5L conditions were not pooled because their ceilings differed by 12.8% points.

### Awareness of repeated questions

Interviewers recorded whether respondents noticed being asked the same questions twice. The repetition was noticed by 39.2% of respondents who answered the EQ-5D-5L first and 43.7% of those who answered the EQ-5D-3L first.

Restricting the analysis to interviewer-present interviews, the EQ-5D-5L ceiling was 34.5% (159 of 461) with no prior EQ-5D-3L exposure, 43.0% (104 of 242) after the EQ-5D-3L among respondents who did not notice the repetition, and 53.2% (100 of 188) among those who did. Relative to no prior EQ-5D-3L exposure, the corresponding differences were 8.5% points (95% CI 0.9 to 16.1) and 18.7% points (10.4 to 27.1) (Table S11).

Respondents who noticed the repetition were older. After adjustment for age, sex, and long-standing illness, noticing the repetition remained associated with the EQ-5D-5L full-health ceiling (adjusted odds ratio 1.71, 95% CI 1.08 to 2.69; Table S12). Because noticing was observed rather than randomised, these estimates should be considered hypothesis-generating.

## Discussion

The early-position comparison reproduced the established descriptive differences between the EQ-5D-3L and EQ-5D-5L: the EQ-5D-5L full-health ceiling was 16.7% points lower and informativity was higher on every dimension. These findings are consistent with comparisons reported in the United Kingdom and internationally [8, 9, 12, 13]. A large United Kingdom matched comparison of more than 1 million GP Patient Survey respondents provides a useful external benchmark [27].

The main contribution is methodological: the apparent magnitude of these between-version differences depended strongly on administration order. The three-arm design separated questionnaire position from prior exposure to the alternative version. For the EQ-5D-5L, most of the observed order effect was associated with prior EQ-5D-3L exposure rather than questionnaire position: position alone changed the ceiling by 2.5% points, whereas prior EQ-5D-3L exposure at the same position raised it by 12.8 points. Because the late-position EQ-5D-5L items were identical in the two conditions, this contrast estimates the effect associated with prior EQ-5D-3L exposure while holding questionnaire position constant. The effect extended beyond the ceiling: response-level distributions shifted, informativity fell for the EQ-5D-5L and rose for the EQ-5D-3L, and the number of distinct EQ-5D-5L profiles fell from 146 to 113.

Previous head-to-head comparisons have not treated administration sequence consistently. In the systematic review by Buchholz et al. [8], EQ-5D-5L was generally administered before EQ-5D-3L in fixed-order studies, while other studies used randomised or crossover designs; two crossover studies reported no influence of administration order on response trends. Janssen et al. [13] administered EQ-5D-5L first specifically because earlier evidence suggested that prior EQ-5D-3L completion could reduce use of the intermediate EQ-5D-5L response levels. Our findings extend this literature by showing that administration-order effects vary across measurement properties. Prior exposure affected the full-health ceiling, response-level distributions and informativity, whereas logical inconsistency remained largely unchanged.

The pattern is consistent with carry-over or contextual priming rather than a generic late-position or fatigue effect [28], although the mechanism cannot be established from these data. Moving the EQ-5D-5L later in the questionnaire without prior EQ-5D-3L exposure produced little change in the ceiling, whereas prior EQ-5D-3L exposure at the same late position produced a much larger shift. The awareness gradient provides additional, although non-randomised, support for a response-contamination mechanism. Our findings extend to descriptive-system comparisons the warning previously raised for mapping: prior exposure to the alternative EQ-5D version can alter subsequent responses and should not be assumed to be neutral [18].

The practical implication is that fixed-order within-person comparisons can attenuate the apparent difference between the two descriptive systems. In these data, prior EQ-5D-3L exposure raised the EQ-5D-5L ceiling, whereas prior EQ-5D-5L exposure lowered the EQ-5D-3L ceiling; the two versions therefore moved towards each other under either fixed sequence. Consistent with this pattern, the paired ceiling difference was 1.3% points when EQ-5D-3L was administered first and 11.6 points when EQ-5D-5L was administered first, compared with 16.7 points in the early-position between-group comparison. The direction and magnitude of any resulting bias in previous head-to-head studies may therefore depend on the sequence used, which should be reported explicitly rather than assumed.

Distinguishing stable between-version measurement differences from administration-induced response contamination is central to interpreting these findings. The early-position comparison provides an estimate of the between-version difference when neither version has been preceded by the other, whereas the late-position EQ-5D-5L contrast estimates the additional effect associated with prior EQ-5D-3L exposure while holding questionnaire position constant. The larger EQ-5D-5L ceiling among respondents observed to notice the repetition is consistent with contamination, but because awareness was not randomised it should be regarded as supportive rather than causal evidence. This distinction also clarifies study design: randomised between-person comparisons avoid within-respondent carry-over, whereas within-person head-to-head studies should randomise or counterbalance administration order and report it explicitly. Generalisation beyond the EQ-5D should be cautious, because randomised studies of other patient-reported outcome measures have found small or inconsistent order effects [29–31]. Such effects may be more pronounced when instruments have highly overlapping content and similar response structures, as the EQ-5D-3L and EQ-5D-5L do, but this hypothesis requires direct testing.

### Strengths and limitations

The principal strength of this study is its randomised-order design within a probability-based general-population panel. Because the descriptive systems occupied fixed questionnaire positions and the late-position EQ-5D-5L items were identical for respondents who had and had not previously answered the EQ-5D-3L, the design permitted questionnaire position and prior exposure to be separated.

Several limitations should be acknowledged. First, the early-position comparison included fewer than 1,000 respondents per version, which is modest relative to the largest published comparisons; subgroup estimates therefore have greater uncertainty. Second, the panel comprised relatively engaged respondents, many with previous panel experience, so the magnitude of the order effect may not generalise to all survey modes or populations. Third, respondents allocated to the EQ-5D-5L-only condition were more likely to have incomplete responses after covariate adjustment, which may have affected the estimated questionnaire-position effect. Fourth, the awareness analysis relied on a non-randomised interviewer observation recorded only in interviewer-present interviews and should therefore be interpreted cautiously. Fifth, experimental condition was assigned at household level, whereas the analyses did not explicitly account for within-household clustering. This may have resulted in some underestimation of uncertainty in the inferential statistics. Finally, respondents who answered the EQ-5D-3L first had completed five additional EQ-5D items before reaching the late position, making this additional response burden inseparable from prior exposure itself.

Further work should test both the mechanism and the consequences of administration order. Re-expressing the order effect in utility terms under the recently published United Kingdom EQ-5D-5L value set would be a natural extension [32, 33], because any order-induced shift in EQ-5D profiles will propagate into EQ-5D values and, potentially, quality-adjusted life-year estimates [21, 34]. Cognitive interviewing and experiments that vary the spacing, visual presentation and mode of repeated instruments could help distinguish response-scale anchoring, consistency effects and response burden. Replication in fully web-based and less survey-experienced samples would establish how far the observed effects generalise. Whether similar effects arise with other patient-reported outcome measures is likely to depend on content overlap and response structure and should be tested rather than assumed.

## Conclusions

In this randomised general-population experiment, administration sequence altered responses to the EQ-5D-3L and EQ-5D-5L. For the EQ-5D-5L, prior EQ-5D-3L exposure at the same questionnaire position increased the full-health ceiling by 12.8% points, whereas questionnaire position alone changed it by 2.5 points. Fixed-order administration attenuated the between-version ceiling difference in either sequence. Administration order also affected response distributions, informativity and the number of distinct EQ-5D profiles. Within-person head-to-head studies should therefore randomise or counterbalance administration order and report the sequence used. 

## Supplementary Information

Supplementary Material 1.

## Data Availability

No datasets were generated or analysed during the current study.

## References

[1] EuroQol Group. (1990). EuroQol–a new facility for the measurement of health-related quality of life. *Health Policy*, *16*(3), 199–208. 10.1016/0168-8510(90)90421-9 10109801

[2] Brooks, R. (1996). EuroQol: the current state of play. *Health Policy*, *37*(1), 53–72. 10.1016/0168-8510(96)00822-6 10158943

[3] Devlin, N. J., & Brooks, R. (2017). EQ-5D and the EuroQol Group: Past, Present and Future. *Applied Health Economics And Health Policy*, *15*(2), 127–137. 10.1007/s40258-017-0310-5 PMC5343080 28194657

[4] Devlin, N. J., Shah, K. K., Feng, Y., Mulhern, B., & van Hout, B. (2018). Valuing health-related quality of life: An EQ-5D-5L value set for England. *Health Economics*, *27*(1), 7–22. 10.1002/hec.3564 PMC6680214 28833869

[5] Dolan, P. (1997). Modeling valuations for EuroQol health states. *Medical Care*, *35*(11), 1095–1108. 10.1097/00005650-199711000-00002 9366889

[6] Herdman, M., Gudex, C., Lloyd, A., Janssen, M., Kind, P., Parkin, D., et al. (2011). Development and preliminary testing of the new five-level version of EQ-5D (EQ-5D-5L). *Quality Of Life Research*, *20*(10), 1727–1736. 10.1007/s11136-011-9903-x PMC3220807 21479777

[7] Bas Janssen, M. F., Birnie, E., & Bonsel, G. J. (2007). Evaluating the discriminatory power of EQ-5D, HUI2 and HUI3 in a US general population survey using Shannon’s indices. *Quality Of Life Research*, *16*(5), 895–904. 10.1007/s11136-006-9160-6 PMC1915610 17294285

[8] Buchholz, I., Janssen, M. F., Kohlmann, T., & Feng, Y. S. (2018). A Systematic Review of Studies Comparing the Measurement Properties of the Three-Level and Five-Level Versions of the EQ-5D. *Pharmacoeconomics*, *36*(6), 645–661. 10.1007/s40273-018-0642-5 PMC5954044 29572719

[9] Cheng, L. J., Pan, T., Chen, L. A., Cheng, J. Y., Mulhern, B., Devlin, N., & Luo, N. (2024). The Ceiling Effects of EQ-5D-3L and 5L in General Population Health Surveys: A Systematic Review and Meta-Analysis. *Value In Health : The Journal Of The International Society For Pharmacoeconomics And Outcomes Research*, *27*(7), 986–997. 10.1016/j.jval.2024.02.018 38467187

[10] Feng, Y. S., Kohlmann, T., Janssen, M. F., & Buchholz, I. (2021). Psychometric properties of the EQ-5D-5L: a systematic review of the literature. *Quality Of Life Research*, *30*(3), 647–673. 10.1007/s11136-020-02688-y PMC7952346 33284428

[11] Craig, B. M., Pickard, A. S., & Lubetkin, E. I. (2014). Health problems are more common, but less severe when measured using newer EQ-5D versions. *Journal Of Clinical Epidemiology*, *67*(1), 93–99. 10.1016/j.jclinepi.2013.07.011 PMC4091801 24075597

[12] Janssen, M. F., Bonsel, G. J., Luo, N. (2018). Is EQ-5D-5L Better Than EQ-5D-3L? A Head-to-Head Comparison of Descriptive Systems and Value Sets from Seven Countries. *Pharmacoeconomics*, *36*(6), 675–697. 10.1007/s40273-018-0623-8 PMC5954015 29470821

[13] Janssen, M. F., Pickard, A. S., Golicki, D., Gudex, C., Niewada, M., Scalone, L., et al. (2013). Measurement properties of the EQ-5D-5L compared to the EQ-5D-3L across eight patient groups: a multi-country study. *Quality Of Life Research*, *22*(7), 1717–1727. 10.1007/s11136-012-0322-4 PMC3764313 23184421

[14] Marten, O., & Greiner, W. (2022). Feasibility properties of the EQ-5D-3L and 5L in the general population: evidence from the GP Patient Survey on the impact of age. *Health Economics Review*, *12*(1), 28. 10.1186/s13561-022-00374-y PMC9121571 35593942

[15] Rencz, F., Brodszky, V., & Janssen, M. F. (2023). A Direct Comparison of the Measurement Properties of EQ-5D-5L, PROMIS-29 + 2 and PROMIS Global Health Instruments and EQ-5D-5L and PROPr Utilities in a General Population Sample. *Value In Health : The Journal Of The International Society For Pharmacoeconomics And Outcomes Research*, *26*(7), 1045–1056. 10.1016/j.jval.2023.02.002 36804583

[16] Pham, M., Craig, B. M., & Rencz, F. (2025). The psychometric performance of the EQ-5D-5L composite and component items in the U.S. General population and by age group. *Quality Of Life Research*, *34*(12), 3649–3661. 10.1007/s11136-025-04053-3 PMC12689771 40911276

[17] Nikl, A., Brodszky, V., Szabó, Á., & Rencz, F. (2026). Comparing the measurement properties of the EQ-5D-Y-3L and EQ-5D-3L in a general population sample of adults. *The European Journal Of Health Economics*, *27*(3), 675–689. 10.1007/s10198-025-01839-7 PMC13190750 41107671

[18] Hernández-Alava, M., & Pudney, S. (2022). Mapping between EQ-5D-3L and EQ-5D-5L: A survey experiment on the validity of multi-instrument data. *Health Economics*, *31*(6), 923–939. 10.1002/hec.4487 PMC9303872 35229404

[19] Hernández-Alava, M., & Pudney, S. (2018). Eq5Dmap: A Command for Mapping between EQ-5D-3L and EQ-5D-5L. *The Stata Journal*, *18*(2), 395–415. 10.1177/1536867X1801800207

[20] van Hout, B., Janssen, M. F., Feng, Y. S., Kohlmann, T., Busschbach, J., Golicki, D., et al. (2012). Interim scoring for the EQ-5D-5L: mapping the EQ-5D-5L to EQ-5D-3L value sets. *Value In Health : The Journal Of The International Society For Pharmacoeconomics And Outcomes Research*, *15*(5), 708–715. 10.1016/j.jval.2012.02.008 22867780

[21] Hernandez Alava, M., Wailoo, A., Grimm, S., Pudney, S., Gomes, M., Sadique, Z., et al. (2018). EQ-5D-5L versus EQ-5D-3L: The Impact on Cost Effectiveness in the United Kingdom. *Value In Health : The Journal Of The International Society For Pharmacoeconomics And Outcomes Research*, *21*(1), 49–56. 10.1016/j.jval.2017.09.004 29304940

[22] Pennington, B., Hernandez-Alava, M., Pudney, S., & Wailoo, A. (2019). The Impact of Moving from EQ-5D-3L to -5L in NICE Technology Appraisals. *Pharmacoeconomics*, *37*(1), 75–84. 10.1007/s40273-018-0701-y 30094806

[23] Chang, C-H., & Reeve, B. B. (2005). Item Response Theory and its Applications to Patient-Reported Outcomes Measurement. *Evaluation & the Health Professions*, *28*(3), 264–282. 10.1177/0163278705278275 16123257

[24] Teresi, J. A., & Fleishman, J. A. (2007). Differential item functioning and health assessment. *Quality of Life Research*, *16*(Suppl 1), 33–42. 10.1007/s11136-007-9184-6 17443420

[25] Penton, H., Dayson, C., Hulme, C., & Young, T. (2022). An Investigation of Age-Related Differential Item Functioning in the EQ-5D-5L Using Item Response Theory and Logistic Regression. *Value In Health : The Journal Of The International Society For Pharmacoeconomics And Outcomes Research*, *25*(9), 1566–1574. 10.1016/j.jval.2022.03.009 35487819

[26] Devlin, N. J., Parkin, D., & Browne, J. (2010). Patient-reported outcome measures in the NHS: new methods for analysing and reporting EQ-5D data. *Health Economics*, *19*(8), 886–905. 10.1002/hec.1608 20623685

[27] Thompson, A. J., & Turner, A. J. (2020). A Comparison of the EQ-5D-3L and EQ-5D-5L. *Pharmacoeconomics*, *38*(6), 575–591. 10.1007/s40273-020-00893-8 32180162

[28] Tourangeau, R., Singer, E., & Presser, S. (2003). Context Effects in Attitude Surveys: Effects on Remote Items and Impact on Predictive Validity. *Sociological Methods & Research*, *31*(4), 486–513. 10.1177/0049124103251950

[29] Novotny, P. J., Dueck, A. C., Satele, D., Frost, M. H., Beebe, T. J., Yost, K. J., et al. (2022). Effects of patient-reported outcome assessment order. *Clinical Trials*, *19*(3), 307–315. 10.1177/17407745211073788 PMC9232855 35088616

[30] McColl, E., Eccles, M., Rousseau, N., Steen, I., Parkin, D., & Grimshaw, J. (2003). From the Generic to the Condition-specific? Instrument Order Effects in Quality of Life Assessment. *Medical Care*, *41*(7), 777–790. 10.1097/00005650-200307000-00002 12835602

[31] Cheung, Y. B., Lim, C., Goh, C., Thumboo, J., & Wee, J. (2005). Order effects: a randomised study of three major cancer-specific quality of life instruments. *Health And Quality Of Life Outcomes*, *3*, 37. 10.1186/1477-7525-3-37 PMC1175098 15927054

[32] Rowen, D., Mukuria, C., Bray, N., Carlton, J., Cooper, S., Longworth, L., et al. (2023). UK Valuation of EQ-5D-5L, a Generic Measure of Health-Related Quality of Life: A Study Protocol. *Value In Health : The Journal Of The International Society For Pharmacoeconomics And Outcomes Research*, *26*(11), 1625–1635. 10.1016/j.jval.2023.08.005 37722593

[33] Rowen, D., Mukuria, C., Bray, N., Carlton, J., Longworth, L., Meads, D., et al. (2026). A United Kingdom Value Set for the EQ-5D-5L. *Value In Health : The Journal Of The International Society For Pharmacoeconomics And Outcomes Research*, *29*(5), 858–869. 10.1016/j.jval.2026.03.008 41887403

[34] Mulhern, B., Feng, Y., Shah, K., Janssen, M. F., Herdman, M., van Hout, B., & Devlin, N. (2018). Comparing the UK EQ-5D-3L and English EQ-5D-5L Value Sets. *Pharmacoeconomics*, *36*(6), 699–713. 10.1007/s40273-018-0628-3 PMC5954043 29476363

