# Supplementary material for: Administration-order effects in head-to-head comparisons of the EQ-5D-3L and EQ-5D-5L descriptive systems: evidence from a randomised general-population panel in Great Britain
Source: Qual Life Res. 2026 Oct 22;35(11):311. doi: 10.1007/s11136-026-04419-1 (PMC13690899; doi:10.1007/s11136-026-04419-1)
Supplement: Supplementary file 1 [file 11136_2026_4419_MOESM1_ESM.docx]

**Online Resource 1**

**Article title:** Administration-order effects in head-to-head comparisons of the EQ-5D-3L and EQ-5D-5L descriptive systems: evidence from a randomised general-population panel in Great Britain

**Journal:** *Quality of Life Research*

**Authors:** Ling Jie Cheng, Xun Li

**Corresponding author:** Ling Jie Cheng, Alice Lee Centre for Nursing Studies, Yong Loo Lin School of Medicine, National University of Singapore, and National Perinatal Epidemiology Unit, Nuffield Department of Women’s & Reproductive Health, University of Oxford. E-mail: Cheng.lingjie@nus.edu.sg / Jeremy.cheng@npeu.ox.ac.uk

**Table S1 Response completeness, proxy codes and item validity by randomised arm**

| **Measure** | **Grouping** | **Group 1, n/N (%)** | **Group 2, n/N (%)** | **Group 3, n/N (%)** | **Chi-squared (df)** | **p** |
| --- | --- | --- | --- | --- | --- | --- |
| First instrument complete | randomised arm | 881/952 (92.5) | 884/961 (92.0) | 889/983 (90.4) | 3.02 (2) | 0.221 |
| Proxy code on first instrument | randomised arm | 0/952 (0.0) | 0/961 (0.0) | 0/983 (0.0) |  |  |
| Proxy code on any EQ-5D slot | randomised arm | 0/952 (0.0) | 0/961 (0.0) | 0/983 (0.0) |  |  |
| EQ-5D-5L complete | EQ-5D-5L administration condition | 881/952 (92.5) | 889/983 (90.4) | 872/961 (90.7) | 3.11 (2) | 0.211 |
| EQ-5D-3L complete | EQ-5D-3L administration condition | 884/961 (92.0) | 853/952 (89.6) |  | 3.26 (1) | 0.071 |
| EQ-5D-5L item valid: Mobility | EQ-5D-5L administration condition | 882/952 (92.7) | 890/983 (90.5) | 875/961 (91.0) | 2.96 (2) | 0.228 |
| EQ-5D-5L item valid: Self-care | EQ-5D-5L administration condition | 882/952 (92.7) | 891/983 (90.6) | 875/961 (91.0) | 2.76 (2) | 0.252 |
| EQ-5D-5L item valid: Usual activities | EQ-5D-5L administration condition | 882/952 (92.7) | 891/983 (90.6) | 875/961 (91.0) | 2.76 (2) | 0.252 |
| EQ-5D-5L item valid: Pain & discomfort | EQ-5D-5L administration condition | 882/952 (92.7) | 891/983 (90.6) | 875/961 (91.0) | 2.76 (2) | 0.252 |
| EQ-5D-5L item valid: Anxiety & depression | EQ-5D-5L administration condition | 881/952 (92.5) | 889/983 (90.4) | 872/961 (90.7) | 3.11 (2) | 0.211 |
| EQ-5D-3L item valid: Mobility | EQ-5D-3L administration condition | 888/961 (92.4) | 859/952 (90.2) |  | 2.85 (1) | 0.091 |
| EQ-5D-3L item valid: Self-care | EQ-5D-3L administration condition | 890/961 (92.6) | 858/952 (90.1) |  | 3.75 (1) | 0.053 |
| EQ-5D-3L item valid: Usual activities | EQ-5D-3L administration condition | 890/961 (92.6) | 858/952 (90.1) |  | 3.75 (1) | 0.053 |
| EQ-5D-3L item valid: Pain & discomfort | EQ-5D-3L administration condition | 891/961 (92.7) | 855/952 (89.8) |  | 5.07 (1) | 0.024 |
| EQ-5D-3L item valid: Anxiety & depression | EQ-5D-3L administration condition | 888/961 (92.4) | 855/952 (89.8) |  | 3.97 (1) | 0.046 |

Groups 1 to 3 are the randomised arms (Arm 1 = EQ-5D-5L early then EQ-5D-3L late; Arm 2 = EQ-5D-3L early then EQ-5D-5L late; Arm 3 = EQ-5D-5L late only) or, where the grouping states an administration condition, the conditions defined in Table 5. Blank cells indicate that the grouping has only two levels. Chi-squared tests compare the measure across the stated grouping. No EQ-5D item in any arm carried the UKHLS proxy code; the 94 proxy interviews in the wave were not randomised to an EQ-5D condition.

**Table S2 Correlates of non-completion of the first EQ-5D instrument encountered**

| **Term** | **Odds ratio** | **95% CI** | **p** |
| --- | --- | --- | --- |
| Male (reference) | 1.00 |  |  |
| Female | 1.00 | 0.73 to 1.36 | 0.981 |
| Age, per year | 1.03 | 1.02 to 1.04 | <0.001 |
| Long-standing illness (reference) | 1.00 |  |  |
| No long-standing illness | 0.49 | 0.36 to 0.67 | <0.001 |
| Degree or higher (reference) | 1.00 |  |  |
| A-level or equivalent | 1.74 | 1.06 to 2.88 | 0.030 |
| GCSE or equivalent | 1.59 | 1.00 to 2.53 | 0.051 |
| Other qualification | 4.36 | 2.61 to 7.29 | <0.001 |
| No qualification | 4.95 | 3.17 to 7.75 | <0.001 |
| CAPI (reference) | 1.00 |  |  |
| CATI (omitted; n = 1) | — | — | — |
| CAWI | 0.10 | 0.06 to 0.18 | <0.001 |
| Arm 1 (EQ-5D-5L first; reference) | 1.00 |  |  |
| Arm 2 (EQ-5D-3L first) | 1.17 | 0.79 to 1.73 | 0.435 |
| Arm 3 (EQ-5D-5L only, late position) | 1.49 | 1.03 to 2.17 | 0.035 |

Logistic regression with non-completion of the first EQ-5D instrument as the outcome. Odds ratios above 1 indicate higher odds of not completing. CI, confidence interval; CAPI, computer-assisted personal interview; CATI, computer-assisted telephone interview; CAWI, computer-assisted web interview. CATI was omitted from the regression coefficient table because only one randomised respondent was interviewed by telephone (Table 1).

**Table S3 Proportion reporting any problem (level 2 or worse) by dimension, early-position responses**

| **Dimension** | **EQ-5D-3L, n/N (%)** | **EQ-5D-5L, n/N (%)** | **Difference, percentage points (95% CI)** | **p** |
| --- | --- | --- | --- | --- |
| Mobility | 139/888 (15.7) | 213/882 (24.1) | −8.5 (−12.2 to −4.8) | <0.001 |
| Self-care | 37/890 (4.2) | 87/882 (9.9) | −5.7 (−8.1 to −3.3) | <0.001 |
| Usual activities | 129/890 (14.5) | 195/882 (22.1) | −7.6 (−11.2 to −4.0) | <0.001 |
| Pain & discomfort | 295/891 (33.1) | 451/882 (51.1) | −18.0 (−22.6 to −13.5) | <0.001 |
| Anxiety & depression | 237/888 (26.7) | 316/881 (35.9) | −9.2 (−13.5 to −4.9) | <0.001 |

Each descriptive system is taken from the randomised arm in which it was answered in the early questionnaire position. Differences are EQ-5D-3L minus EQ-5D-5L, so negative values indicate that more problems were reported on EQ-5D-5L. p values are continuity-corrected Pearson chi-squared. CI, confidence interval.

**Table S4 Distribution of EQ-5D-3L by EQ-5D-5L response combinations across administration sequences**

| **Dimension** | **Paired responses, n** | **Combinations observed** | **Chi-squared** | **df** | **p** | **Permutation p** | **Smallest expected cell** | **Cells expected <5** | **Holm-adjusted p** |
| --- | --- | --- | --- | --- | --- | --- | --- | --- | --- |
| Mobility | 1719 | 20 | 16.75 | 9 | 0.053 | 0.036 | 0.49 | 8 | 0.053 |
| Self-care | 1718 | 22 | 23.29 | 10 | 0.010 | 0.003 | 0.49 | 10 | 0.029 |
| Usual activities | 1718 | 30 | 26.23 | 14 | 0.024 | 0.009 | 0.49 | 18 | 0.048 |
| Pain & discomfort | 1717 | 26 | 91.84 | 12 | <0.001 | <0.001 | 0.49 | 10 | <0.001 |
| Anxiety & depression | 1712 | 28 | 35.79 | 13 | <0.001 | <0.001 | 0.49 | 12 | 0.003 |

Tests assess whether the joint distribution of paired EQ-5D-3L and EQ-5D-5L responses differs between the two administration sequences (EQ-5D-3L first versus EQ-5D-5L first). Permutation p values come from 2,000 random reallocations of the sequence label and are reported because many response combinations are rare. Holm adjustment is applied across the five dimensions.

**Table S5 Logical inconsistency between paired EQ-5D-3L and EQ-5D-5L responses, by dimension and administration sequence**

| **Dimension** | **Pooled, %** | **Pooled n** | **EQ-5D-3L first, n/N (%)** | **EQ-5D-5L first, n/N (%)** | **Difference, percentage points (95% CI)** | **p** | **Holm-adjusted p** |
| --- | --- | --- | --- | --- | --- | --- | --- |
| Mobility | 2.0 | 1719 | 13/868 (1.5) | 22/851 (2.6) | −1.1 (−2.4 to 0.2) | 0.154 | 0.400 |
| Self-care | 1.4 | 1718 | 11/868 (1.3) | 13/850 (1.5) | −0.3 (−1.4 to 0.8) | 0.797 | 0.797 |
| Usual activities | 2.6 | 1718 | 15/868 (1.7) | 30/850 (3.5) | −1.8 (−3.3 to −0.3) | 0.029 | 0.144 |
| Pain & discomfort | 2.6 | 1717 | 28/869 (3.2) | 16/848 (1.9) | 1.3 (−0.1 to 2.8) | 0.110 | 0.400 |
| Anxiety & depression | 2.0 | 1712 | 23/865 (2.7) | 12/847 (1.4) | 1.2 (−0.1 to 2.6) | 0.100 | 0.400 |

Inconsistency is defined following Janssen et al. as a logically incompatible pairing of EQ-5D-3L and EQ-5D-5L levels for the same respondent and dimension, for example, an EQ-5D-3L response of no problems paired with an EQ-5D-5L response of level 3 or worse. Differences are EQ-5D-3L-first minus EQ-5D-5L-first in percentage points. No dimension differed significantly by sequence after Holm adjustment. This table replaces Table S1 of the original submission. CI, confidence interval.

**Table S6 Tests of the level distribution across administration conditions, by dimension**

| **Instrument** | **Dimension** | **Chi-squared** | **df** | **p** | **Permutation p** | **Smallest expected cell** | **Holm-adjusted p** |
| --- | --- | --- | --- | --- | --- | --- | --- |
| EQ-5D-5L | Mobility | 18.28 | 8 | 0.019 | 0.018 | 2.65 | 0.077 |
| EQ-5D-3L | Mobility | 11.25 | 2 | 0.004 | 0.001 | 1.48 | 0.011 |
| EQ-5D-5L | Self-care | 17.67 | 8 | 0.024 | 0.021 | 1.65 | 0.077 |
| EQ-5D-3L | Self-care | 16.79 | 2 | <0.001 | <0.001 | 2.94 | 0.001 |
| EQ-5D-5L | Usual activities | 12.32 | 8 | 0.137 | 0.135 | 4.96 | 0.137 |
| EQ-5D-3L | Usual activities | 12.06 | 2 | 0.002 | 0.003 | 7.85 | 0.010 |
| EQ-5D-5L | Pain & discomfort | 49.45 | 8 | <0.001 | <0.001 | 11.23 | <0.001 |
| EQ-5D-3L | Pain & discomfort | 6.72 | 2 | 0.035 | 0.043 | 27.42 | 0.070 |
| EQ-5D-5L | Anxiety & depression | 15.16 | 8 | 0.056 | 0.055 | 12.54 | 0.112 |
| EQ-5D-3L | Anxiety & depression | 2.01 | 2 | 0.366 | 0.358 | 28.45 | 0.366 |

Pearson chi-squared tests of the distribution of responses across levels by administration condition (three conditions for EQ-5D-5L, two for EQ-5D-3L). Permutation p values come from 2,000 random reallocations of the administration condition and are reported because some expected cells are sparse. Holm adjustment is applied separately across the five dimensions for each version.

**Table S7 Response counts by level and administration condition, by dimension**

| **Instrument** | **Dimension** | **Condition** | **n** | **Level 1** | **Level 2** | **Level 3** | **Level 4** | **Level 5** | **Level 1 (%)** | **Difference vs early (95% CI)** |
| --- | --- | --- | --- | --- | --- | --- | --- | --- | --- | --- |
| EQ-5D-5L | Mobility | EQ-5D-5L early position | 882 | 669 | 103 | 62 | 45 | 3 | 75.8 | Reference |
| EQ-5D-5L | Mobility | EQ-5D-5L late position, no prior EQ-5D-3L | 890 | 681 | 121 | 52 | 32 | 4 | 76.5 | 0.7 (−3.3 to 4.6) |
| EQ-5D-5L | Mobility | EQ-5D-5L late position, after EQ-5D-3L | 875 | 714 | 96 | 42 | 22 | 1 | 81.6 | 5.8 (1.9 to 9.6) |
| EQ-5D-3L | Mobility | EQ-5D-3L early position | 888 | 749 | 138 | 1 |  |  | 84.3 | Reference |
| EQ-5D-3L | Mobility | EQ-5D-3L late position, after EQ-5D-5L | 859 | 671 | 186 | 2 |  |  | 78.1 | −6.2 (−9.9 to −2.6) |
| EQ-5D-5L | Self-care | EQ-5D-5L early position | 882 | 795 | 50 | 25 | 10 | 2 | 90.1 | Reference |
| EQ-5D-5L | Self-care | EQ-5D-5L late position, no prior EQ-5D-3L | 891 | 820 | 39 | 24 | 8 | 0 | 92.0 | 1.9 (−0.8 to 4.5) |
| EQ-5D-5L | Self-care | EQ-5D-5L late position, after EQ-5D-3L | 875 | 827 | 21 | 19 | 5 | 3 | 94.5 | 4.4 (1.9 to 6.9) |
| EQ-5D-3L | Self-care | EQ-5D-3L early position | 890 | 853 | 33 | 4 |  |  | 95.8 | Reference |
| EQ-5D-3L | Self-care | EQ-5D-3L late position, after EQ-5D-5L | 858 | 785 | 71 | 2 |  |  | 91.5 | −4.3 (−6.6 to −2.1) |
| EQ-5D-5L | Usual activities | EQ-5D-5L early position | 882 | 687 | 109 | 57 | 26 | 3 | 77.9 | Reference |
| EQ-5D-5L | Usual activities | EQ-5D-5L late position, no prior EQ-5D-3L | 891 | 702 | 112 | 47 | 21 | 9 | 78.8 | 0.9 (−2.9 to 4.7) |
| EQ-5D-5L | Usual activities | EQ-5D-5L late position, after EQ-5D-3L | 875 | 715 | 103 | 34 | 20 | 3 | 81.7 | 3.8 (0.1 to 7.6) |
| EQ-5D-3L | Usual activities | EQ-5D-3L early position | 890 | 761 | 119 | 10 |  |  | 85.5 | Reference |
| EQ-5D-3L | Usual activities | EQ-5D-3L late position, after EQ-5D-5L | 858 | 686 | 166 | 6 |  |  | 80.0 | −5.5 (−9.1 to −2.0) |
| EQ-5D-5L | Pain & discomfort | EQ-5D-5L early position | 882 | 431 | 283 | 109 | 45 | 14 | 48.9 | Reference |
| EQ-5D-5L | Pain & discomfort | EQ-5D-5L late position, no prior EQ-5D-3L | 891 | 476 | 280 | 92 | 31 | 12 | 53.4 | 4.6 (−0.1 to 9.2) |
| EQ-5D-5L | Pain & discomfort | EQ-5D-5L late position, after EQ-5D-3L | 875 | 563 | 215 | 64 | 25 | 8 | 64.3 | 15.5 (10.9 to 20.1) |
| EQ-5D-3L | Pain & discomfort | EQ-5D-3L early position | 891 | 596 | 265 | 30 |  |  | 66.9 | Reference |
| EQ-5D-3L | Pain & discomfort | EQ-5D-3L late position, after EQ-5D-5L | 855 | 525 | 304 | 26 |  |  | 61.4 | −5.5 (−10.0 to −1.0) |
| EQ-5D-5L | Anxiety & depression | EQ-5D-5L early position | 881 | 565 | 193 | 82 | 30 | 11 | 64.1 | Reference |
| EQ-5D-5L | Anxiety & depression | EQ-5D-5L late position, no prior EQ-5D-3L | 889 | 577 | 178 | 92 | 26 | 16 | 64.9 | 0.8 (−3.7 to 5.2) |
| EQ-5D-5L | Anxiety & depression | EQ-5D-5L late position, after EQ-5D-3L | 872 | 622 | 143 | 74 | 22 | 11 | 71.3 | 7.2 (2.8 to 11.6) |
| EQ-5D-3L | Anxiety & depression | EQ-5D-3L early position | 888 | 651 | 206 | 31 |  |  | 73.3 | Reference |
| EQ-5D-3L | Anxiety & depression | EQ-5D-3L late position, after EQ-5D-5L | 855 | 605 | 223 | 27 |  |  | 70.8 | −2.5 (−6.8 to 1.7) |

Counts of responses at each level by administration condition. Level 1 indicates no problems. Differences are in percentage points against the early-position condition for the same version, with Wald 95% confidence intervals. EQ-5D-3L has three response levels and EQ-5D-5L has five.

**Table S8 Informativity by dimension and administration condition**

| **Instrument** | **Dimension** | **Condition** | **n** | **H′** | **J′** | **Difference in H′ vs early (95% CI)** |
| --- | --- | --- | --- | --- | --- | --- |
| EQ-5D-5L | Mobility | EQ-5D-5L early position | 882 | 0.818 | 0.508 | Reference |
| EQ-5D-5L | Mobility | EQ-5D-5L late position, no prior EQ-5D-3L | 890 | 0.786 | 0.488 | −0.032 (−0.123 to 0.059) |
| EQ-5D-5L | Mobility | EQ-5D-5L late position, after EQ-5D-3L | 875 | 0.654 | 0.407 | −0.164 (−0.255 to −0.073) |
| EQ-5D-3L | Mobility | EQ-5D-3L early position | 888 | 0.441 | 0.401 | Reference |
| EQ-5D-3L | Mobility | EQ-5D-3L late position, after EQ-5D-5L | 859 | 0.538 | 0.490 | 0.098 (0.043 to 0.157) |
| EQ-5D-5L | Self-care | EQ-5D-5L early position | 882 | 0.422 | 0.262 | Reference |
| EQ-5D-5L | Self-care | EQ-5D-5L late position, no prior EQ-5D-3L | 891 | 0.353 | 0.219 | −0.069 (−0.156 to 0.018) |
| EQ-5D-5L | Self-care | EQ-5D-5L late position, after EQ-5D-3L | 875 | 0.275 | 0.171 | −0.147 (−0.236 to −0.054) |
| EQ-5D-3L | Self-care | EQ-5D-3L early position | 890 | 0.187 | 0.170 | Reference |
| EQ-5D-3L | Self-care | EQ-5D-3L late position, after EQ-5D-5L | 858 | 0.302 | 0.275 | 0.115 (0.042 to 0.181) |
| EQ-5D-5L | Usual activities | EQ-5D-5L early position | 882 | 0.753 | 0.468 | Reference |
| EQ-5D-5L | Usual activities | EQ-5D-5L late position, no prior EQ-5D-3L | 891 | 0.738 | 0.459 | −0.015 (−0.105 to 0.072) |
| EQ-5D-5L | Usual activities | EQ-5D-5L late position, after EQ-5D-3L | 875 | 0.649 | 0.403 | −0.104 (−0.195 to −0.013) |
| EQ-5D-3L | Usual activities | EQ-5D-3L early position | 890 | 0.453 | 0.413 | Reference |
| EQ-5D-3L | Usual activities | EQ-5D-3L late position, after EQ-5D-5L | 858 | 0.531 | 0.484 | 0.078 (0.012 to 0.144) |
| EQ-5D-5L | Pain & discomfort | EQ-5D-5L early position | 882 | 1.191 | 0.740 | Reference |
| EQ-5D-5L | Pain & discomfort | EQ-5D-5L late position, no prior EQ-5D-3L | 891 | 1.108 | 0.688 | −0.083 (−0.149 to −0.016) |
| EQ-5D-5L | Pain & discomfort | EQ-5D-5L late position, after EQ-5D-3L | 875 | 0.964 | 0.599 | −0.226 (−0.301 to −0.149) |
| EQ-5D-3L | Pain & discomfort | EQ-5D-3L early position | 891 | 0.744 | 0.677 | Reference |
| EQ-5D-3L | Pain & discomfort | EQ-5D-3L late position, after EQ-5D-5L | 855 | 0.773 | 0.704 | 0.030 (−0.023 to 0.084) |
| EQ-5D-5L | Anxiety & depression | EQ-5D-5L early position | 881 | 1.008 | 0.627 | Reference |
| EQ-5D-5L | Anxiety & depression | EQ-5D-5L late position, no prior EQ-5D-3L | 889 | 1.013 | 0.629 | 0.005 (−0.079 to 0.086) |
| EQ-5D-5L | Anxiety & depression | EQ-5D-5L late position, after EQ-5D-3L | 872 | 0.895 | 0.556 | −0.114 (−0.203 to −0.032) |
| EQ-5D-3L | Anxiety & depression | EQ-5D-3L early position | 888 | 0.684 | 0.622 | Reference |
| EQ-5D-3L | Anxiety & depression | EQ-5D-3L late position, after EQ-5D-5L | 855 | 0.704 | 0.641 | 0.021 (−0.040 to 0.084) |

H′ is Shannon’s index and J′ is Shannon’s evenness, calculated at dimension level. Differences in H′ are relative to the early-position condition for the same version, with percentile confidence intervals from 1,000 bootstrap replicates stratified by randomised arm. CI, confidence interval.

**Table S9 Ceiling difference between the two versions under each fixed administration order**

| **Estimate** | **n** | **EQ-5D-3L ceiling, %** | **EQ-5D-5L ceiling, %** | **Difference, percentage points (95% CI)** | **McNemar chi-squared** | **p** | **Design** |
| --- | --- | --- | --- | --- | --- | --- | --- |
| Early-position comparison (independent randomised groups) |  | 52.1 | 35.4 | 16.7 (12.2 to 21.3) |  |  | unpaired between-person |
| EQ-5D-3L first (Arm 2) | 863 | 52.0 | 50.8 | 1.3 (−0.4 to 2.9) | 1.89 | 0.170 | paired (McNemar) |
| EQ-5D-5L first (Arm 1) | 846 | 46.8 | 35.2 | 11.6 (9.2 to 13.9) | 82.53 | <0.001 | paired (McNemar) |

The first row is the between-person estimate using each descriptive system as answered in the early questionnaire position. The remaining rows are within-person estimates among respondents who answered both versions in the stated sequence, tested with McNemar’s test. Both fixed-order estimates are smaller than the early-position difference. CI, confidence interval.

**Table S10 Effect of pooling across administration conditions on between-version differences in full-health ceiling effects**

| **Estimate** | **EQ-5D-3L, n/N (%)** | **EQ-5D-5L, n/N (%)** | **Difference, percentage points (95% CI)** | **Percentage of early-position difference** |
| --- | --- | --- | --- | --- |
| Early-position comparison (independent randomised groups) | 461/884 (52.1) | 312/881 (35.4) | 16.7 (12.2 to 21.3) | 100.0 |
| Pooled within-person arms 1 and 2 | 860/1737 (49.5) | 754/1753 (43.0) | 6.5 (3.2 to 9.8) | 38.8 |
| Pooled all available data | 860/1737 (49.5) | 1091/2642 (41.3) | 8.2 (5.2 to 11.2) | 49.1 |

Pooling combines responses from different administration conditions, as would occur if order were not recorded. The final column expresses the pooled difference as a percentage of the early-position difference of 16.7 percentage points. The two late-position EQ-5D-5L conditions are shown separately elsewhere because their ceilings differ materially. CI, confidence interval.

**Table S11 EQ-5D-5L ceiling by prior exposure to the EQ-5D-3L and awareness of the repetition**

| **Condition** | **Ceiling, n/N (%)** | **95% CI** | **Difference vs no prior EQ-5D-3L, percentage points (95% CI)** | **p** |
| --- | --- | --- | --- | --- |
| No prior EQ-5D-3L (Arm 3) | 159/461 (34.5) | 30.1 to 38.8 | Reference |  |
| After EQ-5D-3L, repetition not noticed | 104/242 (43.0) | 36.7 to 49.2 | 8.5 (0.9 to 16.1) | 0.033 |
| After EQ-5D-3L, repetition noticed | 100/188 (53.2) | 46.1 to 60.3 | 18.7 (10.3 to 27.1) | <0.001 |

Restricted to interviewer-present interviews so that interview mode is held constant. Awareness is an interviewer observation of whether the respondent noticed being asked the same questions twice; it was recorded only for respondents who received both versions. Differences are relative to no prior EQ-5D-3L exposure. CI, confidence interval.

**Table S12 Adjusted association between awareness of the repetition and the EQ-5D-5L ceiling**

| **Term** | **Odds ratio** | **95% CI** | **p** |
| --- | --- | --- | --- |
| Did not notice (reference) | 1.00 |  |  |
| Noticed the repetition | 1.71 | 1.08 to 2.69 | 0.021 |
| Age, per year | 1.00 | 0.99 to 1.01 | 0.880 |
| Male (reference) | 1.00 |  |  |
| Female | 0.93 | 0.60 to 1.45 | 0.751 |
| Long-standing illness (reference) | 1.00 |  |  |
| No long-standing illness | 10.97 | 6.61 to 18.20 | <0.001 |

Logistic regression among respondents who answered EQ-5D-5L in the late position after EQ-5D-3L, restricted to interviewer-present interviews. The outcome is reporting full health on EQ-5D-5L. Awareness was observed rather than randomised, so the association should not be interpreted causally. CI, confidence interval.
